# Barriers and Facilitators to Staying Smoke-Free after Having a Baby, a Qualitative Study: Women’s Views on Support Needed to Prevent Returning to Smoking Postpartum

**DOI:** 10.3390/ijerph182111358

**Published:** 2021-10-28

**Authors:** Lucy Phillips, Katarzyna Anna Campbell, Tim Coleman, Michael Ussher, Sue Cooper, Sarah Lewis, Sophie Orton

**Affiliations:** 1 School of Medicine, University of Nottingham, Nottingham NG7 2RD, UK; lucy.phillips1@nottingham.ac.uk (L.P.); kasia.campbell@nottingham.ac.uk (K.A.C.); tim.coleman@nottingham.ac.uk (T.C.); sue.cooper@nottingham.ac.uk (S.C.); sarah.lewis@nottingham.ac.uk (S.L.); 2 Population Health Research Institute, St. George’s University of London, London SW17 0RE, UK; mussher@sgul.ac.uk; 3 Institute for Social Marketing and Health, University of Stirling, Stirling FK9 4LA, UK

**Keywords:** smoking, pregnancy, relapse, postpartum

## Abstract

Background: Postpartum return to smoking (PPRS) is a common and important public health problem. Interventions to prevent PPRS have not been shown to be effective. We aimed to qualitatively explore the barriers and facilitators to staying smoke-free after having a baby, and women’s views on the support needed to avoid PPRS to inform future intervention development. Methods: We conducted semi-structured telephone interviews (*n* = 26) with pregnant women who quit smoking (*n* = 9), and postpartum women who were abstinent at delivery and returned to smoking (*n* = 7) or stayed smoke-free (*n* = 10). Inductive thematic analysis was used. Results: Five overarching themes were identified: (i) smoking intentions; (ii) facilitators to staying smoke-free; (iii) barriers to staying smoke-free; (iv) support to avoid relapse; and (v) e-cigarettes, nicotine replacement therapy, and varenicline. Facilitators to staying smoke-free were the health benefits to their baby, whilst barriers included stress, cravings, and being in environments where they would previously have smoked. Women wanted continuous offers of support to stay smoke-free throughout the extended postpartum period, with a particular interest in support for partners to quit smoking and self-help support. Women expressed safety concerns for e-cigarettes, nicotine replacement therapy, and varenicline. Conclusions: Offers of support to stay smoke-free should continue throughout the postpartum and engage with partners or other household members who smoke. Reassuring women about the relative safety of nicotine replacement therapy and e-cigarettes by a health professional, particularly for those who are breastfeeding, could be beneficial.

## 1. Introduction

Finding effective ways to help pregnant women quit smoking and remain abstinent in the long term is an important public health priority. In England, in 2019–2020, 12.1% of women were current smokers at 10 weeks gestation [1]. Pregnancy is a life event which strongly motivates women to stop smoking; approximately half of women who smoke before pregnancy manage to stop by childbirth [2]. Regrettably, most women who stop smoking in pregnancy re-start once the baby is born. This relapse is substantial; among women who became abstinent after using cessation support during pregnancy, 43% are smoking again 6 months postpartum [3]. Most pregnant women are ‘spontaneous quitters’ who do not use cessation support, and among this group, relapse rates reached up to 76% within two years of giving birth [4,5,6,7,8].

Reducing maternal smoking would be of significant economic and social benefit by improving the health of both women and their children [9], and is likely to be cost-effective [10,11,12]. Maternal smoking is the primary source of infant and child second-hand smoke exposure [13], a substantial cause of ill health and mortality [9], and children of smoking mothers are twice as likely to become smokers themselves [14]. Whilst there are effective interventions to help women stop smoking during pregnancy [15,16,17], interventions targeted to prevent postpartum relapse to smoking have not been shown to be effective [18].

The development of effective interventions to prevent postpartum return to smoking (PPRS) requires understanding of women’s experiences of returning to smoking postpartum, and their views on the support needed. A previous qualitative systematic review [19] of this topic comprised studies conducted in the US or Canada, which may not be generalizable elsewhere, and were carried out prior to the popularization of e-cigarettes (ECs) [20]. In the UK, ECs were used in 29% of quit attempts in 2021 [21] and around 5% of women reported using ECs in pregnancy [22]; however, little is known about the role that ECs could play in PPRS. We are aware of only one other qualitative study conducted since the systematic review; a UK study [23] sought women’s views on the support they would like to receive to prevent PPRS, and found that women preferred an intervention delivered by a ‘credible source’, and intervention components, such as tailored self-help, partner involvement, and social support. However, views on ECs were mixed [23]. This study was limited to one geographical location within the UK and was the first to explore the use of ECs for PPRS prevention. Further research is needed to validate and build on these findings.

This study is a UK-based qualitative exploration of the barriers and facilitators to staying smoke-free after having a baby. We also explored women’s views on support needed, including pharmacological interventions and ECs, to prevent returning to smoking postpartum, and the best timing of this support.

## 2. Materials and Methods

We conducted a qualitative study using semi-structured telephone interviews, incorporating a longitudinal approach where pregnant participants were invited to be re-interviewed after giving birth to explore any changes in views between pregnancy and postpartum. Ethical approval was granted by the Faculty of Medicine and Health Science Research Ethics Committee, the University of Nottingham (Reference number: 89-1808).

### 2.1. Inclusion and Exclusion Criteria

Women were eligible for the study if they were over 16 years of age, smoked prior to pregnancy and were either: (i) currently pregnant and abstinent from smoking (self-reported quitting during early pregnancy or in the 3 months prior to pregnancy); or (ii) postpartum (less than 12 months) and self-reported, quitting in either early pregnancy or in the 3 months prior to pregnancy, and were abstinent at the time of birth.

Women were excluded if they were unable to understand the study procedure sufficiently to provide consent, or if they were unable to read or understand the study procedures in English or participate in an interview in English.

### 2.2. Recruitment

A purposive sampling strategy was used to recruit three target groups; (1) pregnant women who had quit smoking; (2) postpartum women who had returned to smoking; and (3) postpartum women who had remained smoke-free. We aimed to interview up to 10 women in each group, or until reaching data saturation.

Participants were recruited online via Facebook banner adverts targeted towards females aged 16–45, and IP addresses located in the UK. The advert linked to a screening questionnaire hosted by Jisc Online Surveys [24], with brief information about the study; women were asked to provide pregnancy status (pregnant or postpartum and months postpartum), smoking during pregnancy and postpartum, and contact details. Eligible women were sent a participation information sheet and consent form via email and contacted by a researcher approximately 48 h later. Women gave verbal consent for participation over the telephone prior to the interview and confirmed consent for the recording.

Women who were pregnant at the time of interview gave optional consent to be contacted after the birth for a second interview. Using self-reported due dates, women were contacted when their babies were >4 weeks old and invited to complete an interview following the same procedure as previously.

### 2.3. Interviews

Three experienced, female researchers conducted the interviews (S.O.: Ph.D., health psychology background, non-smoker, K.C.: Ph.D., health psychology background, non-smoker, L.P.: MSc, health psychology background, non-smoker). The interviewers introduced themselves as researchers from the University of Nottingham. Prior to interviewing, researchers recorded participants’ weeks gestation or infant age, maternal age, education, occupation, relationship status, smoking status, and cigarettes per day prior to pregnancy and cigarettes per day if currently smoking. Interviews were audio-recorded and transcribed verbatim by a transcriber external to the research team. Interviewees received a shopping voucher worth GBP 20 as compensation for their time.

Interview topic guides were semi-structured covering the following topics: smoking behavior in pregnancy, motivations for quitting smoking, support accessed and strategies used to quit smoking, barriers and facilitators to staying abstinent during pregnancy and postpartum, social influences, smoking intentions and views on using nicotine replacement therapy (NRT), varenicline (a medication that can aid smoking cessation), and ECs. Second interviews conducted with women after they had given birth covered the same topics, with additional questions on changes to their previous views and experiences, their experience of returning to smoking if applicable, and new barriers and facilitators to staying smoke free. Interviews lasted 30–60 min.

Participant patient involvement engagement was utilized throughout the study, providing feedback on study aims, materials, methods, and recruitment procedures.

### 2.4. Analysis

The data were analyzed using inductive thematic analysis. This approach allows themes and patterns within data to be identified, interpreted, organized, and described [25]. The researchers familiarized themselves with the data through reading and re-reading transcripts and systematically noting initial codes and patterns across the data. These were then collated into potential themes and subthemes, with all examples of the themes within the data gathered. Next, these themes were reviewed, ensuring they reflected the coded extracts and the entire data set. The themes were then further refined, giving clear definitions and names for each theme.

Analysis was led by one researcher (L.P.), and coding was checked by two other researchers (S.O., K.C.). Analysis was facilitated using NVivo 12 software which was used to organize, structure, and code the themes [26]. Coding was initially derived from the two postpartum groups of women. Once data saturation was achieved, responses from pregnant women were coded using the initial coding structure. At this point, it was noted that there was little difference in the themes between the pregnant and postpartum groups of women; any minor differences were then highlighted within each theme. The coding structure was discussed between the researchers at each stage and was refined to group relevant codes into themes. All members of the research team inputted, reviewed, and refined the final themes. Once a final coding structure was agreed, the dataset was recoded using the agreed coding frame.

### 2.5. Reflexive Note

This study was conducted from the epistemological stance of critical realism, which assumes that experiences are understood through human interpretation and mediated by our beliefs and perceptions [27]. It is therefore important to consider how the authors’ beliefs and experiences may have affected the research process. All authors are researchers with backgrounds in public health, primary health care, or psychology, and have extensive experience in conducting research in the field of smoking cessation in pregnancy and postpartum. The researchers are aware of the dangers of smoking in pregnancy and postpartum and hold the belief that using pharmacotherapy or ECs is less harmful than smoking. This study is UK-based, where pregnant women have access to stop smoking services, which support the use of NRT and ECs during pregnancy and postpartum to aid smoking cessation [28]. To maintain rigor and minimize any bias that this may introduce, authors remained aware of their views and made efforts to remain objective throughout the research.

## 3. Results

A total of 123 women were eligible and interested in taking part; 80 were contacted and 26 consented to an interview. Interviews were conducted between November 2019 and January 2020. At the time of interview, 9 women were pregnant and smoke free, 10 were postpartum and still smoke-free, and 7 were postpartum and had returned to smoking. Five of the women interviewed during pregnancy consented to a postpartum follow-up interview. Participants were aged 19 to 38 years and the majority were either married or cohabiting (Table 1).

Five overarching themes were identified: (i) smoking intentions; (ii) facilitators to staying smoke free; (iii) barriers to staying smoke free; (iv) support to avoid relapse; and (v) e-cigarettes, nicotine replacement therapy, and varenicline.

### 3.1. Smoking Intentions

The majority of women wanted to remain smoke-free after having their baby. Reasons for this focused on protecting the health of their baby, their own health, financial benefits of quitting, and having succeeded in being smoke-free for so long during their pregnancy.

*“At first, the only reason I was quitting smoking was because of the fact I was pregnant. So obviously I had planned just to continue to smoke, and have a smoke-free house after pregnancy. But after being quit smoking for so long, like, I don’t miss smoking. And I don’t see the reason of going back to smoking just because I was smoking before pregnancy. So I do plan to just continue to be completely smoke free.”*

(P021 pregnant non-smoker)

During pregnancy, women generally did not intend to return to their pre-pregnancy smoking behaviors after childbirth; although, a couple of women did comment that they did not have strong intentions to stay smoke-free in case they found it too difficult to remain abstinent. Some talked about returning to smoking but with measures to protect their baby from second-hand smoke exposure, such as only smoking outside or occasional smoking when they were not with their baby.

*“I mean I feel like if I was on a night out, like socially, like I wouldn’t see a problem. As long as I wasn’t like, smoking near the baby, but like it wouldn’t hurt the baby, it would only be me.”*

(P044 pregnant non-smoker)

Some pregnant women lacked confidence in their ability to maintain their abstinence after having their baby, describing being unable to predict how they may feel towards smoking in the future, and past experience of relapses.

*“I don’t know. If I’m being honest, I don’t know, because I quit before and I went back to it. Whether I’ll ever go back full time, whether I can ever afford to smoke full time again, I don’t know. But I can’t, I wouldn’t want to say “No, I will never pick up a cigarette again in my life” because I don’t know if I will.”*

(P035 pregnant non-smoker)

### 3.2. Facilitators to Staying Smoke Free

Protecting the health of their baby and wanting to raise their baby in a smoke-free environment were the primary motivators given by both pregnant and postpartum women for remaining abstinent in the long term.

*“I think it’s more for like my baby and like keeping him healthy. Like when I was pregnant I didn’t want to hurt him by smoking and like I don’t want to like have him like breathing in loads of smoke now.”*

(P044 Postpartum non-smoker)

Women stressed that they did not want their baby to smell of cigarettes, or to be able to smell cigarettes on their clothes when they were holding them.

*“I don’t want to like breathe cigarette smoke over her. I don’t want her clothes to smell like cigarettes and I don’t want her to like hug me and smell cigarettes, I don’t think that’s very nice.”*

(P071, postpartum non-smoker)

Breastfeeding was a facilitator for staying smoke-free. A minority of women reported that breastfeeding did not impact their smoking; however, most were uncomfortable smoking if they were breastfeeding due to concerns about nicotine passing into their breastmilk or smelling of smoke whilst feeding their baby. Some women who relapsed described smoking less whilst they were breastfeeding.

*“I don’t want him having nicotine, I know nicotine comes in through your breastmilk, not only that, I don’t want to be cuddling into him and sleeping next to him in bed and, you know, he’s got a next to me cot if I’m stinking of cigarette smoke.”*

(P001, postpartum non-smoker)

For pregnant women, pregnancy symptoms, such as sickness and heightened sense of smell, put them off smoking or being around others that smoked. Postpartum women also reported aversions towards the smell of cigarettes after being abstinent for so long, and some who had smoked postpartum (either lapse or relapse) described not enjoying smoking as much as before pregnancy.

*“Because I don’t have that stress that I had before and when I smoke sometimes I can’t even finish it. It’s like it doesn’t have any pleasure, I don’t find any pleasure like before.”*

(P039 postpartum smoker)

The financial benefit of quitting and being able to put the money saved towards things for their baby or a family holiday was a prominent facilitator to staying smoke free.

*“If for example if a packet of cigarettes is £9 or a pouch of tobacco is £25 for example, I could buy nappies with that or I could buy her toys or clothes and things like that. I thought of everything else I could spend the money on rather than that.”*

(P069, postpartum non-smoker)

### 3.3. Barriers to Staying Smoke Free

Both groups of postpartum women discussed triggers for returning to smoking including stress and cravings. Experiences of stress were described both generally and the stress of being a parent.

*“I think it’s also the stress of having the two children rather than just the one as well, because obviously now life is double busy, so every time that I do get stressed out with either one of them, that’s the first thing that I want to do [smoke], even though I do stop myself.”*

(P041 postpartum smoker)

Most of the postpartum women reported cravings for cigarettes after having their baby, although experiences of cravings differed slightly between the two postpartum groups. Some of the women who had returned to smoking reported increased cravings (either before or after returning to smoking), whereas most women who had remained smoke-free mentioned milder cravings or feeling like they had ‘forgotten to do something’, but did not experience cravings so strong that they wanted to smoke again.

*“Occasionally, yeah, occasionally. I’d say I’ve probably got to the point now where they’re very few and far between, but still, no, yeah, I do get them, especially if I smell it.”*

(P063 postpartum non-smoker)

Additionally, struggles in dealing with nicotine addiction was mentioned by one of the women who had returned to smoking postpartum.

*“The addiction, the nicotine addiction. I don’t think the nicotine addiction ever really goes, because I think you have go like a year or two to give up smoking before you’re fully away from it. I heard it was something like that. But I don’t think the addiction ever really goes. You just learn to manage whilst pregnant.”*

(P019 postpartum smoker)

Social environments were a barrier to remaining smoke-free, such as returning to work with colleagues who smoked, socializing with friends, and drinking alcohol. Postpartum women described avoiding these environments or avoiding alcohol so that they were not tempted to smoke, whereas pregnant women discussed what aspects of socializing they could foresee as being difficult after having their baby.

*“I do worry about if I have a drink. I haven’t had a drink since he’s been born. I don’t intend to, but I think that’s when I’ll struggle most.”*

(P001 postpartum follow-up non-smoker)

*“I think social situations will be difficult, especially summer months. Me and my friends go out quite a lot during the summer months, a lot of beer gardens, you know we go on a lot of walks, we go and sit in a pub for a bit and then walk back and whatnot. That’s the hard part”*

(P035 pregnant)

A couple of women talked about adapting to their new role and identity as a mother, describing how smoking helped to remind them of who they were before they became a mother.

*“One of the things as well, which I found really odd, is that it reminds me of before I was a Mum. So now I feel like my whole identity has changed and it’s all I’m about now is these babies, which is wonderful and I love it, I love being a Mum, but at the same time when I’m doing that it reminds me a bit of how it was before, when I was just my own person”*

(P046 postpartum follow-up smoker)

Women in the two postpartum groups also felt there was a lack of postpartum healthcare support, with no support past the standard health visitor appointments and no enquiries into smoking status of women after having the babies or offers of cessation support.

*“I think because if you’re not smoking in pregnancy I feel like I don’t see, they don’t really help you after pregnancy. I think they just see it as a great thing that you’ve stopped for being pregnant and there’s like a kind of presumption that you won’t start again.”*

(P017 postpartum smoker)

### 3.4. Support to Avoid Relapse

We sought women’s views on support needed to avoid returning to smoking. Women described the importance of social support from partners, family, and friends to remain smoke-free. Their partners not smoking was particularly important for women’s abstinence, with many wishing that there was more support from health care professionals to encourage partners to quit.

*“They tell the mums about the dangers of smoking and everything else, but, they don’t really say a lot to your partner, they say “you ought to not smoke”, but really they need to talk to both people, I think, ‘cause you’re both picking up the baby and, you know, it’s second hand smoke and things like…or third hand smoke they call it now, don’t they?”*

(P058 postpartum non-smoker)

There was interest in relapse prevention support delivered by a health professional, and frustration that there was not more support offered postpartum. Women preferred this to be delivered by either health visitors, due to their close and frequent contact with families, or smoking cessation advisors. Women from all three groups stressed the need for continuous offers of support after having their baby, with many describing how visits from health care professionals, and therefore conversations about smoking, tended to stop if the baby was feeding and putting on weight.

*“I think the main thing is keeping that support, not just after 3 months, I think it should be on-going. If you want the support it should be there for you. Rather than just giving up on after 3 months.”*

(P041 postpartum smoker)

*“I think a general check in would be useful yeah. Once you’ve had your baby, apart from the health visitor, it’s very sort of baby focused. You’re sort of left on your own with it a bit aren’t you? And you know it’s just as damaging for the baby to be a smoker after it’s born as it is before it’s born. So it does seem a bit silly that nobody’s sort of interested after that.”*

(P045 pregnant non-smoker)

Women were less interested in group support sessions as these were perceived as not being easily accessible whilst caring for a newborn. However, some did feel ‘peer support’ from other mothers who had an experience of smoking cessation would be useful, and a few suggested social support through online forums or ‘mum groups’ via social networking sites.

*“I would probably look on my phone instead or connect with people through Facebook. I’m in a couple of mum groups, a cloth nappy group and a couple of other groups, so I’d probably get on there and have a little chat and a catch up with people.”*

(P063 postpartum non-smoker)

Women talked about a variety of different self-help methods they had tried, both during and after pregnancy, and how helpful they found them. Mobile phone apps, both general and cessation-specific, were popular as they were easily accessible, and cessation-specific apps helped to reinforce motivation for quitting smoking.

*“And I had an app when I got pregnant, I downloaded like a smoke-free app and it would tell you how long you haven’t smoked for and you could put your intensity of your cravings into it, and stuff, and I’ve still got that app now and it’s nice to just check and see how long you’ve actually done it for.”*

(P061 postpartum non-smoker)

### 3.5. E-Cigarettes, Nicotine Replacement Therapy and Varenicline

We sought women’s views on using ECs, NRT, and varenicline to prevent smoking relapse after having their baby. There was a lack of awareness of varenicline as a medication that could aid smoking cessation or prevent smoking relapse postpartum. Whilst there was more awareness for ECs and NRT, some women were not aware that they were able to use these whilst breastfeeding.

*“I’ve not used any nicotine replacement or anything like that, and I don’t think I can, actually, as much so with breastfeeding my daughter.”*

(P058 postpartum non-smoker)

Safety concerns were common. Most women who had heard of varenicline described knowing other people who had used it and had negative or dangerous side effects, which had deterred them from using the medication themselves.

*“I know someone that was on them and they had really bad side effects with them, like really, really bad”*

(P061, postpartum non-smoker)

Women expressed safety concerns, particularly about ECs, citing the lack of research on their safety and long-term health consequences, and reports that they were as harmful as smoking tobacco.

*“I think it was very unclear just now whether vaping is as bad for you as smoking or if it is not. There’s a lot of mixed information out there about ‘oh well, it’s just air, it’s just vape, it’s not got any nicotine in it, it just waves around, it can’t do any harm to you’ and then there’s other things that say ‘well, it causes popcorn lungs, it’s just as bad as smoking, it’s just as addictive’. So the information that’s out there I don’t think is clear.”*

(P001, pregnant)

Women were reluctant to use EC and NRT due to a concern that they would be continuing their addiction to nicotine, and that they would be unable to end that addiction. Women described examples of people they knew who had quit smoking using ECs or NRT, and continued to use these in the long term, swapping one nicotine addiction for another.

*“I just…well, I kind of wanted to beat the addiction really. So, by replacing it with something else that provides you with nicotine, it’s not really kicking the addiction.”*

(P067 postpartum non-smoker)

Despite these concerns over using varenicline, NRT, and ECs, many women would be more willing to try these to avoid returning to smoking after having their baby if they were recommended by a health professional.

*“If it was a trained healthcare professional it would have more weight with me and I probably would consider using them.”*

(P064, postpartum non-smoker)

## 4. Discussion

Women described that the main facilitator to staying smoke-free after having their baby was the health benefits to their baby, whilst barriers included stress, cravings, and being in environments where they would previously have smoked. Women were keen for ongoing offers of support to stay smoke-free from health professionals after childbirth, with a particular interest in support for partners to quit smoking and self-help support. Women expressed safety concerns for ECs, NRT, and varenicline used to prevent relapse back to smoking; however, they said they would find these more acceptable if they were recommended by a health professional.

### 4.1. Strengths & Limitations

A key strength of our study is its novelty; few UK studies have qualitatively explored the views of women on the kind of support needed to help prevent PPRS, in particular their views towards ECs, NRT, and varenicline. Recruitment occurred online using Facebook banner adverts, which allowed researchers to identify and interview women from across the UK. Using this recruitment method, we were able to identify postpartum women who were no longer under the care of maternity health services and therefore explore women’s views and experiences throughout the first year postpartum. Few previous qualitative studies have also explored these issues outside of the initial postpartum period. Furthermore, three experienced qualitative researchers conducted the interviews and analyses with a rigorous and systematic approach, enhancing the reliability of our findings.

There were some limitations. Our online recruitment methods would not have reached women who do not have internet access or use Facebook as a social media platform. This study was conducted in the UK, and so may not be applicable elsewhere. Our sample was older, more well educated, and more likely to be in a relationship than is typically associated with smoking in pregnancy [29]; however, this is reflective of the characteristics of women who are more likely to quit smoking during pregnancy [30], suggesting that our sample is broadly representative of this population.

### 4.2. Comparison to Previous Studies

The barriers and facilitators to remaining smoke-free after having a baby that we identified were similar to those found in a previous qualitative systematic review [19]. They also closely related to factors identified as predictors of PPRS in a quantitative systematic review [31], including living with partners or household members who smoke, experiencing higher stress, not breastfeeding, and having low confidence in remaining abstinent postpartum. Our findings show that these are similarly applicable to a UK population.

Our study supports earlier research [23] that women are interested in being offered ongoing support to remain smoke-free during pregnancy and after having their baby. Despite this, very few women who quit smoking when pregnant report accessing support after having their baby [32]. This may be because support is not routinely offered postpartum; women in our study reported fewer offers of cessation support and fewer conversations with health professionals about their smoking postpartum than during pregnancy; however, as interest remains high, it is important to continue support throughout the extended postpartum.

There was a preference for support to be offered by health professionals, such as health visitors or smoking cessation advisors. Women expressed a particular interest in support for partners to quit smoking. Partner smoking is commonly identified as a barrier to remaining smoke-free postpartum [19,23,31]; however, there are few effective cessation or relapse prevention interventions that engage with partners or target partner smoking [33,34]. Future interventions to prevent postpartum smoking relapse should support all household members who smoke. Similar to our study, previous research on interest towards smoking cessation support in pregnancy and early postpartum found that women were interested in self-help, such as booklets, websites, and phone apps, and perceived these kinds of support as being most useful [32]. This is likely to reflect the ease with which self-help can be accessed from home and can fit around the demands of caring for a baby. Our findings have identified novel opportunities to incorporate social support in self-help interventions, for example using social media.

Women in our study were cautious about using ECs, NRT, or varenicline to prevent relapse. Previous research similarly found women were concerned about using ECs to prevent smoking postpartum due to ‘continuing addiction’ and concerns about using ECs whilst breastfeeding [23]. Our findings also reflect those from studies carried out in pregnancy; a Cochrane review on determinants of NRT and EC use in pregnancy similarly reported that women had concerns about their safety for the baby and potential addictiveness [35]. Our findings build on previous research by showing that these concerns continue into the postpartum and highlight a lack of awareness of the safety of ECs and NRT whilst breastfeeding. Less than 5% of recent ex-smoking women use NRT during the first 3 months postpartum [32]; however, ECs and NRT could be useful for women struggling to manage their cravings, which was a commonly cited barrier to staying smoke-free in this study. Within the few women who were aware of varenicline, views on possibly using this were negative, and, as varenicline is not recommended whilst breastfeeding, this might not be appropriate for some women.

## 5. Conclusions

The findings confirm previous observations that offers of support to stay smoke-free, as well as engagement with partners or other household members who smoke, should continue throughout the postpartum. Reassuring women about the relative safety of NRT and ECs by a health professional, particularly for those who are breastfeeding, could be beneficial.

## Figures and Tables

**Table 1:** Participant characteristics.

Participant ID	Weeks’ Gestation/Months Postpartum	Age	Relationship Status	Any Other Children	Highest Educational Qualification	Currently Smoking
Pregnant participants						
P045	13 weeks	36	Other	2	Foundation Degree	No
P007	16 weeks	31	Cohabiting	1	GCSE	No
P044	19 weeks	21	Single	No	Degree	No
P024	21 weeks	30	Cohabiting	No	Degree	No
P035	22 weeks	35	Married	No	A-Level	No
P001	23 weeks	31	Cohabiting	1	Diploma	No
P046	24 weeks	24	Cohabiting	No	Masters	No
P027	33 weeks	34	Cohabiting	3	AS Level	No
P021	36 weeks	20	Single	No	Level 3	No
Pregnant participants re-interviewed postpartum						
P044 (follow-up interview)	6 weeks postpartum	21	Cohabiting	No	Degree	No
P001 (follow-up interview)	9 weeks postpartum	31	Cohabiting	1	Diploma	No
P027 (follow-up interview)	14 weeks postpartum	34	Cohabiting	3	AS Level	No
P021 (follow-up interview)	15 weeks postpartum	20	Single	No	Level 3	No
P046 (follow-up interview)	10 weeks postpartum	25	Cohabiting	No	Masters	Yes
Postpartum participants						
P067	4 months postpartum	34	Cohabiting	No	PGCE	No
P056	4.5 months postpartum	28	Cohabiting	No	Diploma	No
P014	5.5 months postpartum	38	Cohabiting	No	Masters	No
P058	5.5 months postpartum	36	Cohabiting	1	Higher National Diploma	No
P083	6 months postpartum	22	Cohabiting	No	Diploma	No
P063	6.5 months postpartum	37	Cohabiting	No	NVQ Level 2	No
P069	6.5 months postpartum	27	Married	No	NVQ	No
P064	8 months postpartum	27	Married	No	A-Level	No
P074	10 months postpartum	35	Married	6	GCSE	No
P071	11 months postpartum	28	Cohabiting	3	GCSE	No
P019	<1 month postpartum	19	Married	No	A-Level	Yes
P061	4 months postpartum	22	Cohabiting	No	GCSE	Yes (Occasionally)
P077	6.5 months postpartum	24	Cohabiting	No	Higher National Certificate	Yes
P039	7 months postpartum	26	Cohabiting	No	GCSE	Yes
P041	7.5 months postpartum	26	Married	1	NVQ Level 3	Yes
P017	9 months postpartum	29	Single	1	Degree	Yes
P068	12 months postpartum	20	Single	No	A-Level	Yes (Occasionally)

## Data Availability

All data sharing requests can be made to S.O.
